# Abiotic versus Biotic Drivers of Ocean pH Variation under Fast Sea Ice in McMurdo Sound, Antarctica

**DOI:** 10.1371/journal.pone.0107239

**Published:** 2014-09-15

**Authors:** Paul G. Matson, Libe Washburn, Todd R. Martz, Gretchen E. Hofmann

**Affiliations:** 1 Department of Ecology, Evolution, and Marine Biology, University of California Santa Barbara, Santa Barbara, California, United States of America; 2 Department of Geography, University of California Santa Barbara, Santa Barbara, California, United States of America; 3 Geosciences Research Division, Scripps Institution of Oceanography, University of California San Diego, La Jolla, California, United States of America; The Evergreen State College, United States of America

## Abstract

Ocean acidification is expected to have a major effect on the marine carbonate system over the next century, particularly in high latitude seas. Less appreciated is natural environmental variation within these systems, particularly in terms of pH, and how this natural variation may inform laboratory experiments. In this study, we deployed sensor-equipped moorings at 20 m depths at three locations in McMurdo Sound, comprising deep (bottom depth>200 m: Hut Point Peninsula) and shallow environments (bottom depth ∼25 m: Cape Evans and New Harbor). Our sensors recorded high-frequency variation in pH (Hut Point and Cape Evans only), tide (Cape Evans and New Harbor), and water mass properties (temperature and salinity) during spring and early summer 2011. These collective observations showed that (1) pH differed spatially both in terms of mean pH (Cape Evans: 8.009±0.015; Hut Point: 8.020±0.007) and range of pH (Cape Evans: 0.090; Hut Point: 0.036), and (2) pH was not related to the mixing of two water masses, suggesting that the observed pH variation is likely not driven by this abiotic process. Given the large daily fluctuation in pH at Cape Evans, we developed a simple mechanistic model to explore the potential for biotic processes – in this case algal photosynthesis – to increase pH by fixing carbon from the water column. For this model, we incorporated published photosynthetic parameters for the three dominant algal functional groups found at Cape Evans (benthic fleshy red macroalgae, crustose coralline algae, and sea ice algal communities) to estimate oxygen produced/carbon fixed from the water column underneath fast sea ice and the resulting pH change. These results suggest that biotic processes may be a primary driver of pH variation observed under fast sea ice at Cape Evans and potentially at other shallow sites in McMurdo Sound.

## Introduction

Information regarding natural environmental variation is crucial to understanding how marine populations may respond to future changes in ocean climate. Recent technological advances, such as the development of deployable pH sensors [1], have enabled marine scientists to begin to explore natural variation in ocean pH at much higher temporal frequencies and provided a glimpse at how natural pH variation differs across ecosystems [2]. The magnitude of this pH variation between ecosystems is readily apparent, with coastal Antarctic and offshore oligotrophic ecosystems exhibiting much lower magnitudes of variation than seen in coastal ecosystems in temperate and tropical regions [2]. However, we have less understanding of how pH variation may differ within geographic regions and the relative strength of abiotic (*e.g.,* surface air/sea mixing, subsurface water mass mixing, heat flux) and biotic (*e.g.,* photosynthesis and respiration) processes in driving these patterns. Such spatial heterogeneity in pH variation could lead to some areas functioning as either hot-spots for adaptation to dynamic environmental conditions or refugia from future conditions.

Polar ecosystems are expected to be the first to experience the impacts of anthropogenic induced climate change, as well as to experience the greatest relative change in environmental conditions [3]. Antarctic marine ecosystems are generally recognized for their environmental stability; for instance, ocean temperature in the southern Ross Sea remains at or near −1.8°C for the majority of the year [4], [5]. This homogeneous environment is reflected in the physiology of Antarctic species (reviewed by [6]), with many being stenothermal and demonstrating poor abilities to acclimate to elevated temperatures [7] and some having even lost the ability to generate a heat shock response [8]. While the majority of ecophysiological research with Antarctic marine species has focused on temperature stress, much less is known regarding how these species may respond to fluctuations in the pH environment (see [9]–[13]). However, recent data have suggested that some critical species may respond in a deleterious fashion [14], [15]. Since marine organisms are adapted to local conditions [16], [17], it is crucial to improve our understanding of natural ocean pH variation within Antarctic ecosystems in order to more effectively predict how these species may respond to a changing ocean climate.

**Figure 1:**
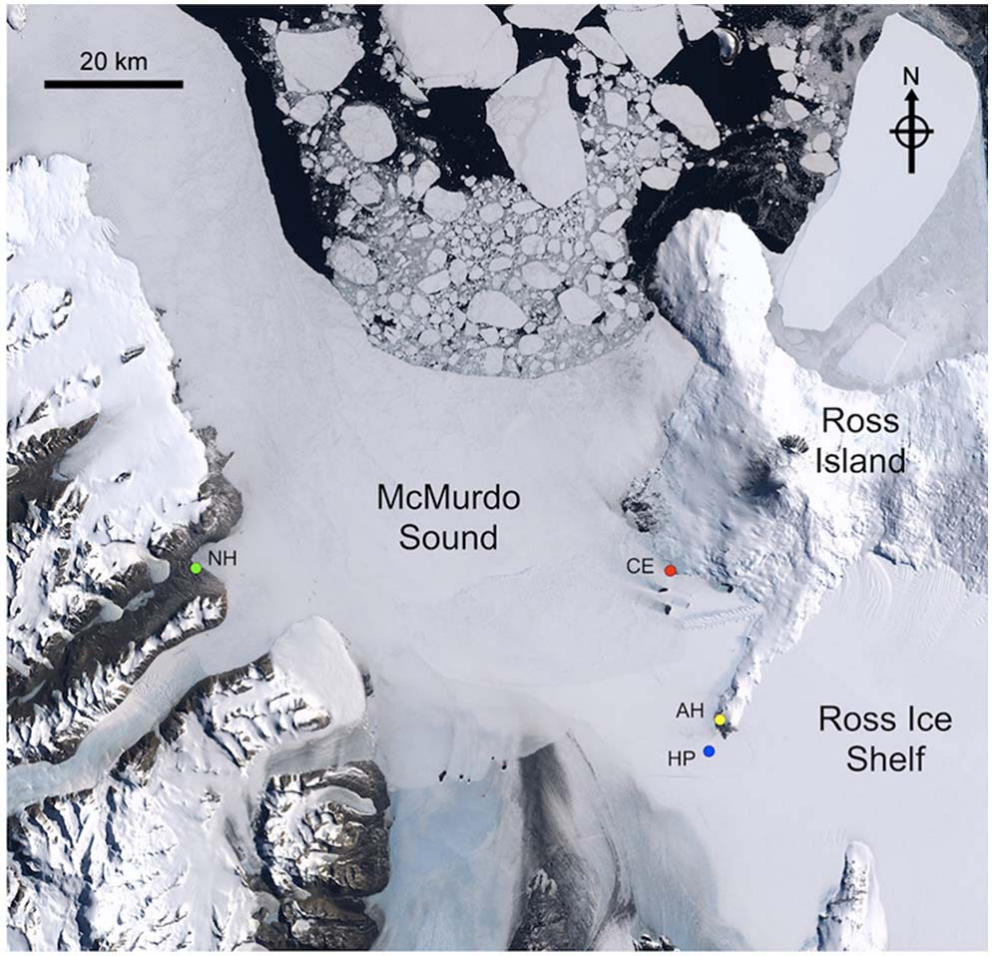
Map showing study location in McMurdo Sound, Antarctica. Colored circles indicate mooring (Hut Point “HP”, blue; Cape Evans “CE”, red; New Harbor “NH”, green) and irradiance sensor locations (Arrival Heights “AH”, yellow). This image is based on Landsat satellite images collected during a previous year and does not reflect sea ice extent from the 2011/2012 season.

Given the remoteness and logistical difficulties inherent to polar research, substantially less is known of the environmental variability of coastal Antarctic seas compared to those in temperate regions. There is a critical need to increase oceanographic observations within the Southern Ocean to improve our understanding of how it will respond to global change [18]. In the southern Ross Sea, McMurdo Sound has received consistent study of its physical and biological oceanography since first discovered by James Ross in 1841. More recent oceanographic investigations have identified two primary water masses present within McMurdo Sound: High Salinity Surface Water (HSSW) and Ice Shelf Water (ISW). These water masses can be identified based on their physical characteristics, with HSSW having relatively warmer temperatures and higher salinity than the colder, fresher ISW [19]. Circulation patterns in the eastern Sound are complex with variation between southward flowing HSSW along Ross Island and northward flowing ISW from under the Ross Ice Shelf, though flow is generally southward [5], [20]–[24] (Figure 1). In the western Sound, current flow is northward and primarily composed of ISW [20], [21], [24], [25]. The physical oceanography within the Sound has created distinct benthic communities on the western and eastern sides of the Sound [21], with greater overall benthic primary production in the east [21], [26]. Coastal bathymetry in the eastern Sound tends to be steeper than in the west, with more abundant hard substrate in the form of basaltic gravel and larger bedrock as opposed to soft glacial dust in the western Sound [27]. Physical scouring by surface sea ice, as well as formation of anchor ice, results in strong zonation patterns with longer-lived species occurring at greater depths [28]. Despite the high latitude of McMurdo Sound (77°S), benthic communities may contain up to three species of macroalgae [27]. These species appear to be adapted to conditions of low temperature and long periods with little or no available light and remain photosynthetically active and capable of rapid responses in production once light levels increase during austral spring [29], [30]. In addition, a diverse microalgal community is present within the bottom of the sea ice [31]. This community may contribute 20–65% of the primary productivity in areas covered by sea ice [32], [33] but is patchily distributed across space and time [34]. While many of these studies have quantified *in* *situ* photosynthetic efficiency and production by algal groups in McMurdo Sound, their contribution to ocean pH variation is not currently known.

A combination of mooring-based ocean observations and modeling was used to explore the natural dynamics of ocean pH during spring and early summer within McMurdo Sound and the potential abiotic and biotic drivers of that variation. To identify patterns of pH variation and potential abiotic drivers, oceanographic sensors were deployed on moorings at multiple locations in McMurdo Sound to observe pH and water mass properties during spring. To identify the potential for a biological driver of variation, specifically under-ice algal photosynthesis, a mechanistic model was developed based on parameters of light availability, photosynthetic efficiency, and estimates of biomass for three algal groups found at Cape Evans on the west coast of Ross Island. This approach helped shed light on the natural dynamics of ocean pH under Antarctic sea ice and what mechanisms may be driving the observed patterns of variation across space.

## Methods

### Study sites

Three oceanographic moorings were deployed within McMurdo Sound during austral spring (October–December) in 2011. Two locations, Cape Evans (S 77° 38.060′, E 166° 24.918′) and Hut Point (S 77° 52.425′, E 166° 35.164′), were located on the eastern side of the sound, while a third location was located on the western side of the sound at New Harbor (S 77° 34.576′, E 163° 31.702′) (Figure 1). This research was conducted under the auspices of the U.S. Antarctic Program in accordance with environmental regulations laid out in the Antarctic Treaty. No deployments were made in protected areas and no permissions or permits were required. These locations were selected in order to observe the two primary water masses within McMurdo sound: the southerly flowing water mass along the eastern side (Cape Evans), the northward flowing water mass coming from under the Ross Ice Sheet (New Harbor), as well as a location where the two water masses may mix at the southern end of Ross Island (Hut Point). The three locations differed in depth, with Cape Evans and New Harbor being shallower coastal sites (<30 m depth) while the Hut Point site was a deeper site (>200 m depth). Benthic moorings were suspended above the seafloor by a subsurface buoy at Cape Evans and New Harbor while the mooring at Hut Point was suspended from the surface through a hole in the ice by a steel cable within a wooden hut. All sensors were deployed at 20 m depth, with the exception of pressure sensors at Cape Evans and New Harbor, which were attached to the mooring anchor.

### Sensor arrays

Moorings were instrumented with a suite of sensors to record time series of temperature, salinity, pH, and tidal height. Temperature and salinity were measured using a non-pumped conductivity-temperature (CT) MicroCAT sensor (SBE-37 SM; Sea-Bird Electronics) that sampled at 5-min intervals. pH was measured using an autonomous data logger based on a Honeywell Durafet pH sensor [1] and sampled at 1-hr intervals. Tidal heights were measured using water level loggers (Hobo U20-001-03-Ti; Onset) that recorded water pressure at 10-min intervals. All oceanographic data was processed using a 1-hr low pass filter and then sampled at 1-hr intervals. All three locations were equipped with CT and pressure sensors while pH sensors were only deployed at Cape Evans and Hut Point; the pH sensor intended for New Harbor was damaged during shipping and rendered inoperable.

Calibration of pH sensors required a discrete water sample collected *in* *situ*. This single point calibration approach is justified when the sensor obeys the Nernst equation and the temperature component of the standard potential has been previously characterized; both of which have been repeatedly demonstrated for these sensors [1]. The water sample was collected adjacent to the sensor by SCUBA divers (Cape Evans) or by lowering a 5 L Niskin sampling bottle from the surface (Hut Point) prior to retrieval. From this sample, a 500 mL water sample was returned to the laboratory for CO_2_ analysis modified from Standard Operating Procedures (SOP) for spectrophotometric pH (SOP 6b) and Total Alkalinity (TA, SOP 3b) [35] as reported in [36]. *In situ* pH was then calculated using CO_2_calc [37] using the constants of [38] as refit by [39]. Due to the calibration approach used, sensor accuracy depends mostly upon collection of a representative discrete sample. Based on experience, there is an expectation that the data presented here accurately represent pH variability with a finite yet unquantified error in accuracy dominated by sampling errors. Past experience suggests that sampling errors lead to vicarious calibration errors of ∼0.01 pH or less. Second order errors due to extending the fit of temperature dependent equilibrium constants in CO_2_calc and temperature dependent sensor calibration coefficients for the SeaFET sensor, both fit to data above zero, introduces additional unquantified error; yet this error is most likely smaller than the aforementioned discrete sampling error [1].

### Photosynthesis-Irradiance Model

A simple mass balance model was constructed to estimate the potential contribution of photosynthesis by sea ice and benthic algae to the pH variation observed at Cape Evans. The control volume for this model comprised four depth bins of equal length and width but varying depths (10 m, 15 m, 20 m, and 25 m) to account for the sloping bathymetry at Cape Evans [27]. These depths determined both the light transmitted to the benthos (z_w_) as well as the volume of the water column. While no measurements of flow from Cape Evans were collected during this study, the water column is generally well-mixed during spring in McMurdo Sound [5], [20] and at Cape Evans in particular [27], likely driven by diurnal tides in the region [40], [41]. Both O_2_ and CO_2(aq)_ were assumed to be uniformly distributed at all times within each bin and no diffusion between bins.

Levels of photosynthetically active radiation (PAR, in units of µmol photons m^−2^ s^−1^) at the surface (*E*_s_) were measured nearby at the NSF UV Monitoring Station (http://uv.biospherical.com/) at McMurdo Station on Arrival Heights (77°50′ S, 166°40′ E). From these data, we identified the day of maximum PAR observed (November 11, 2011; 1495 µmol photons m^−2^ s^−1^) and used this to create a synthetic hourly surface PAR (*E*_s_) dataset for a 24 h period using the equation:
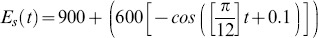
(1)where *t* is hour of the day. PAR at different depths *E*(z,t) was calculated at water depths z below the base of the sea ice at times *t* over a 24 h period using the following equation from [42]:
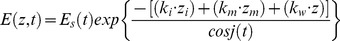
(2)where *k*_i_, *k*_m_, and *k*_w_ are the attenuation coefficients of the sea ice, sea ice-associated microbial layer, and water column, respectively; *z*_i_ and *z*_m_ are the thicknesses of the sea ice and sea ice associated microbial community, respectively. Attenuation of light by the sea ice-associated microbial layer is assumed to be driven primarily from light absorption by sea ice algae; therefore *k*_m_ and *z*_m_ quantify the absorption and concentration of chlorophyll *a* (chl*a*), respectively. Cape Evans has been noted as having little to no snow accumulation at the surface [27], [29], [43] or platelet ice present beneath the sea ice [27], [44], therefore neither were included in the equation. The fraction of incident light transmitted through the sea ice was approximated by:
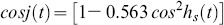
(3)where *h*_s_ is the solar elevation angle above the horizon [45], [46].

Using these estimates of light availability at depth, oxygen production by an algal community composed of sea ice algae, benthic macroalgae, and crustose coralline algae was calculated. For each algal group, we estimated net primary production of oxygen based on the hyperbolic tangent equation of [47]:
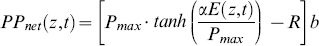
(4)where *P*_max_ is the maximum production at saturating irradiance expressed as oxygen production per unit biomass [µmol O_2_ (g biomass)^−1^ h^−1^], α is the rate of photosynthetic efficiency at non-saturating irradiance per unit biomass [µmol O_2_ (g biomass)^−1^ h^−1^ (µmol photons m^−2^ s^−1^)^−1^], *R* is algal respiration (µmol O_2_ (g biomass)^−1^ h^−1^) per unit biomass, and *b* is the biomass density (g biomass m^−2^). *P*_max_, α, *R*, and *b* for benthic algae used in this model were derived from *in* *situ* measurements of oxygen production at Cape Evans [29], [30]. *P*_max_, α, *R*, and *b* for sea ice algae, originally reported in terms of C [42], were converted to units of O_2_ by multiplying *PP*_net_ (in units of C) by the photosynthetic quotient, estimated as 1.03 (mol O_2/_mol C) [48]. For all taxa, the amount of oxygen produced was divided by the photosynthetic quotient to estimate the total carbon removed from the system. Additionally, all algae in this system were assumed to rely on diffusive uptake of CO_2(aq)_, which has no effect on alkalinity. While the carbon concentration mechanisms for these species are not explicitly known, diffusive uptake of CO_2(aq)_ has been shown to be common for many species of red marine macroalgae from the class *Florideophyceae* (of which *P. antarctica* is a member) [49], as well as in some Antarctic sea ice algae when [CO_2(aq)_]>5 µM [50].

### Carbonate System Estimation

Carbonate system parameters were estimated using CO2SYS for Matlab [51] for both mooring data sets and estimating the effect of photosynthesis on pH. Total alkalinity (TA) was estimated from *in* *situ* salinity and temperature measurements from Hut Point (2336.5±1.1 µmol kgSW^−1^) and Cape Evans (2341.2±0.5 µmol kgSW^−1^) using the equations of [52] for the Southern Ocean. These estimates were in agreement with measurements of alkalinity collected during this study at Hut Point (2335.7±11.8 µmol kgSW^−1^; *n* = 35) and Cape Evans (2342.5±11.3 µmol kgSW^−1^; *n* = 6). To estimate the photosynthesis-irradiance model, a value for total inorganic carbon (C_T_) of 2247.5 µmol kgSW^−1^ was used as a baseline level for the system without photosynthesis based on the lowest pH values recorded at Cape Evans (7.987) during this study and the estimated alkalinity. This value falls in line with winter end-member values of C_T_ from other studies of the carbonate system in Antarctic waters [53]–[56]. Throughout these results, pH is reported on the total hydrogen ion scale. All calculations within CO2SYS use the dissociation constants of [38], refit by [39]. Estimates of total silicate (56 µmol kgSW^−1^) and total phosphate (2.35 µmol kgSW^−1^) were based on values reported values for spring within this region of McMurdo Sound [20], [57]. Salinity and temperature values were 34.7 and −1.9°C, respectively.

## Results

### Mooring Observations

**Figure 2:**
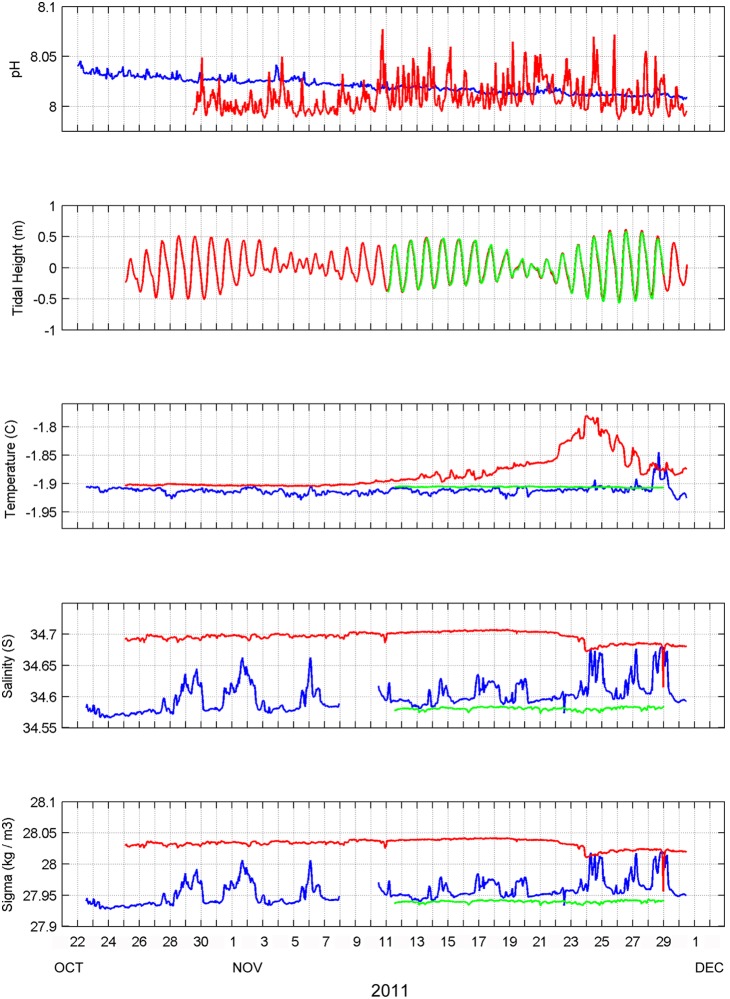
Water mass properties observed during spring in McMurdo Sound. Locations are indicated by color: Hut Point (blue), Cape Evans (red), and New Harbor (green).

Overall patterns of pH variation during spring appeared to vary between locations (Figure 2; Table 1). At Hut Point, pH exhibited very low hourly variation with an overall trend of decreasing pH over the deployment duration. Mean pH was 8.020±0.007 (± s.d.) and ranged from 8.006 to 8.042 and the distribution of these values had a low level of skewness (Pearson’s median skewness: γ  = 0.07). At Cape Evans, pH was initially lower but observed hourly variation was much greater than at Hut Point. Mean pH was 8.009±0.015 and ranged from 7.987 to 8.077 with the distribution showing a higher level of skewness towards higher pH values (Pearson’s median skewness: γ  = 0.25). The mean absolute rate of change in pH also differed between the two locations, with a greater mean rate of change observed at Cape Evans (0.007) versus Hut Point (0.001).

**Table 1:** Descriptive statistics of oceanographic observations from each location.

Variable	Location	Median	Mean	s.d.	Min	Max
pH	Hut Point	8.012	8.020	0.007	8.006	8.042
	Cape Evans	8.005	8.009	0.015	7.987	8.077
Temperature (°C)	Hut Point	−1.913	−1.912	0.007	−1.929	−1.845
	Cape Evans	−1.890	−1.879	0.030	−1.905	−1.781
	New Harbor	−1.906	−1.906	0.001	−1.909	−1.904
Salinity	Hut Point	34.596	34.600	0.023	34.566	34.679
	Cape Evans	34.699	34.697	0.009	34.615	34.708
	New Harbor	34.581	34.581	0.002	34.573	34.585
Density (kg m^−3^)	Hut Point	27.952	27.955	0.019	27.928	28.019
	Cape Evans	28.035	28.033	0.008	27.957	28.042
	New Harbor	27.940	27.940	0.002	27.943	27.933

The number of observations (*n*) varies between sites (Hut Point: for Temperature and pH, *n* = 935, for Salinity and Density, *n* = 875; Cape Evans: *n* = 768; New Harbor: *n* = 420).

**Figure 3:**
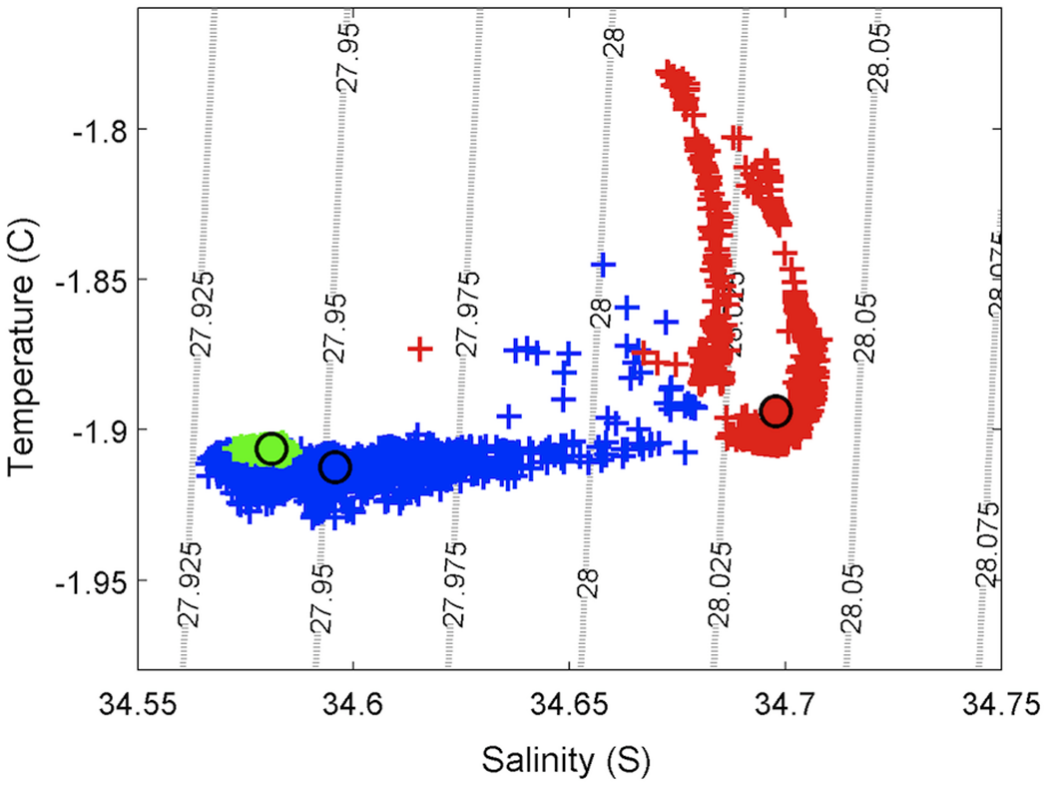
Temperature-Salinity plot showing relationships between temperature, salinity, and density for Hut Point (blue), Cape Evans (red), and New Harbor (green). Individual sampling points are represented by crosses while location-specific median values are represented by filled circles.

During the course of the deployment, three spring tides and two neap tides were observed, with a maximum tidal range of ∼1 m (Figure 2). Water temperature was relatively constant at all locations from late October to late November, with the exception of a warming event at Cape Evans that occurred over a 7 day period in which temperature increased ∼0.12°C (Figure 2; Table 1). Salinity differed on the east and west sides of McMurdo Sound, with greater salinity at Cape Evans versus New Harbor, while salinity at Hut Point fluctuated intermittently throughout the deployment period (Figure 2; Table 1). These fluctuations appear to be mixing between the water masses observed at New Harbor and Cape Evans, as indicated by the relationship between temperature and salinity at the three locations (Figure 3). Seawater density in this region is primarily driven by differences in salinity. Assuming that median values of salinity at Cape Evans and New Harbor represent end-member values for the upper 20 m, calculations indicate that the water mass at Hut Point was composed of ∼14% of the southward flowing water mass mixed with the water mass emerging from under Ross Ice Shelf off Hut Point during this time period. A density discontinuity was observed at Cape Evans on 23–24 November that may indicate advection of a new water mass into the study area or a separate region-specific process. This event appeared to have had little effect on pH at either Hut Point or Cape Evans, with no apparent changes in pH coinciding with density fluctuations at either location. These results suggest that physical processes inherent to water mass advection and mixing do not drive the observed pH variation in McMurdo Sound.

**Figure 4:**
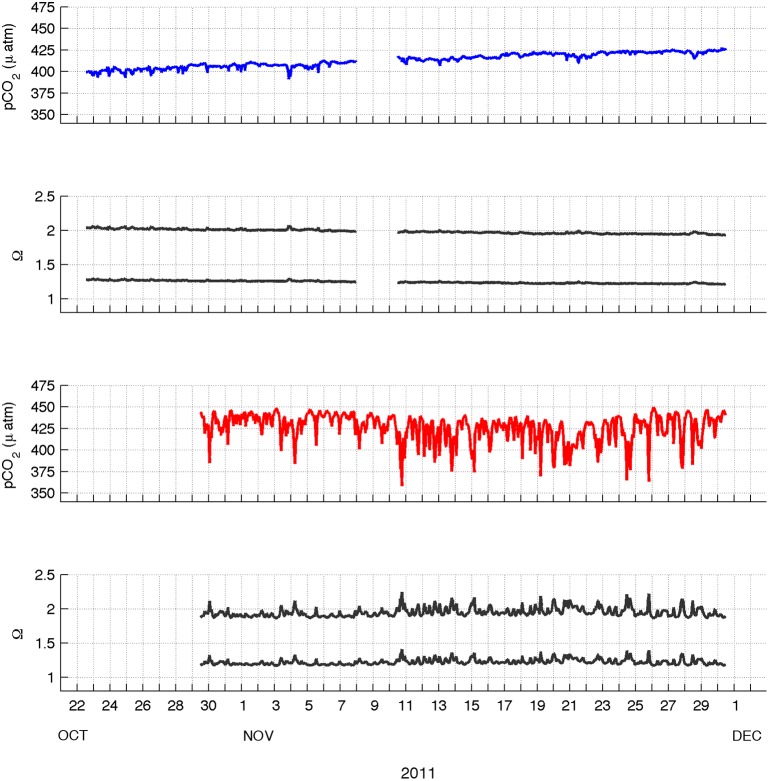
Estimated pCO_2_ and carbonate ion saturation states (Ω) based on observed pH and estimated alkalinity. Data in the upper two panels refer to Hut Point while the two lower panels refer to Cape Evans. Lines in pCO_2_ panels are color coded by location (Hut Point, blue; Cape Evans, red) while lines in carbonate saturation panels indicate Ω_calcite_ (upper black line in panel) and Ω_aragonite_ (lower black line in panel). Alkalinity estimates were location-specific and based on temperature and salinity measurements using the method of [52].

To explore how the calcification environment in McMurdo Sound may vary through time, the carbonate system at both Hut Point and Cape Evans was estimated. Patterns of estimated pCO_2_ fluctuation were inversely related to observed pH, with low levels of variation at Hut Point and higher levels of variation at Cape Evans (Figure 4). At Hut Point, mean pCO_2_ was estimated at 413±8 µatm (± s.d) with a range of 391 to 427 µatm and mean Ω_calcite_ and Ω_aragonite_ of 1.98±0.03 and 1.24±0.02, respectively. At Cape Evans, mean pCO_2_ was estimated at 426±16 µatm with a range of 358 to 450 µatm and mean Ω_calcite_ and Ω_aragonite_ of 1.95±0.06 and 1.22±0.04, respectively. At no time was Ω_aragonite_<1 at either location.

**Figure 5:**
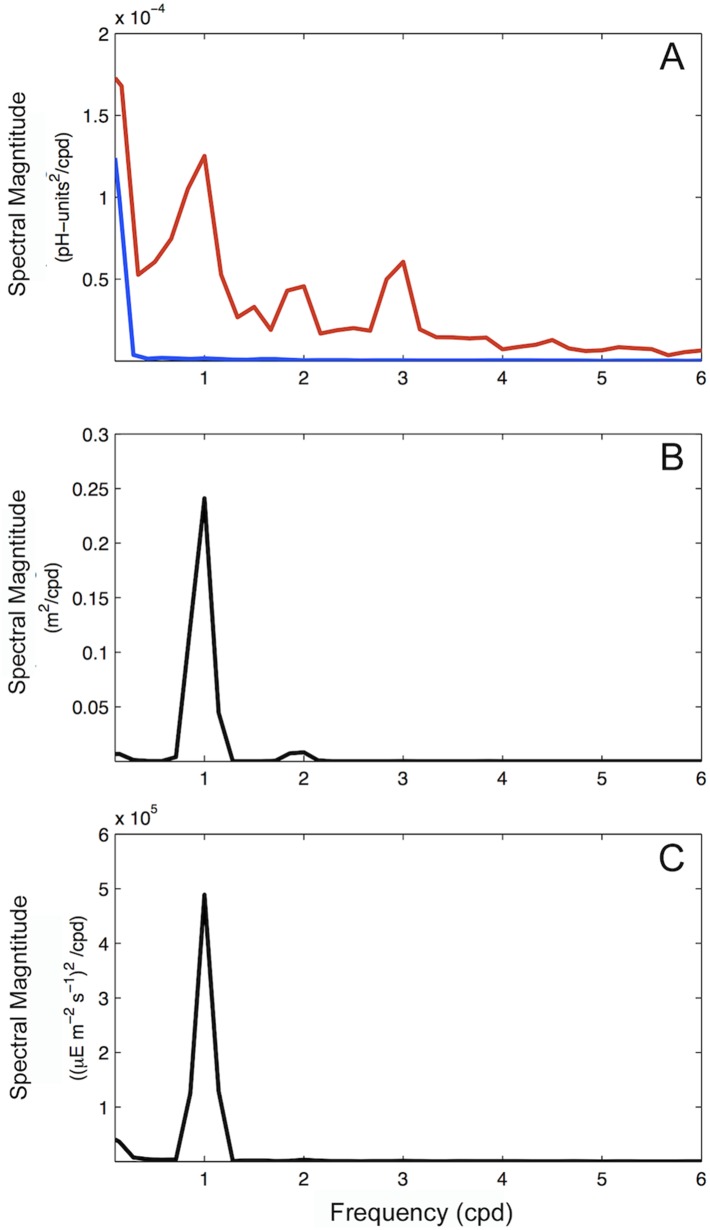
Spectra for pH, tide, and surface irradiance from eastern McMurdo Sound. pH spectra (A) for Hut Point (blue) and Cape Evans (red). Tide spectra (B) are from Cape Evans. Surface irradiance spectra (C) were measured at Arrival Heights. Frequency is in the units of cycles per day.

**Figure 6:**
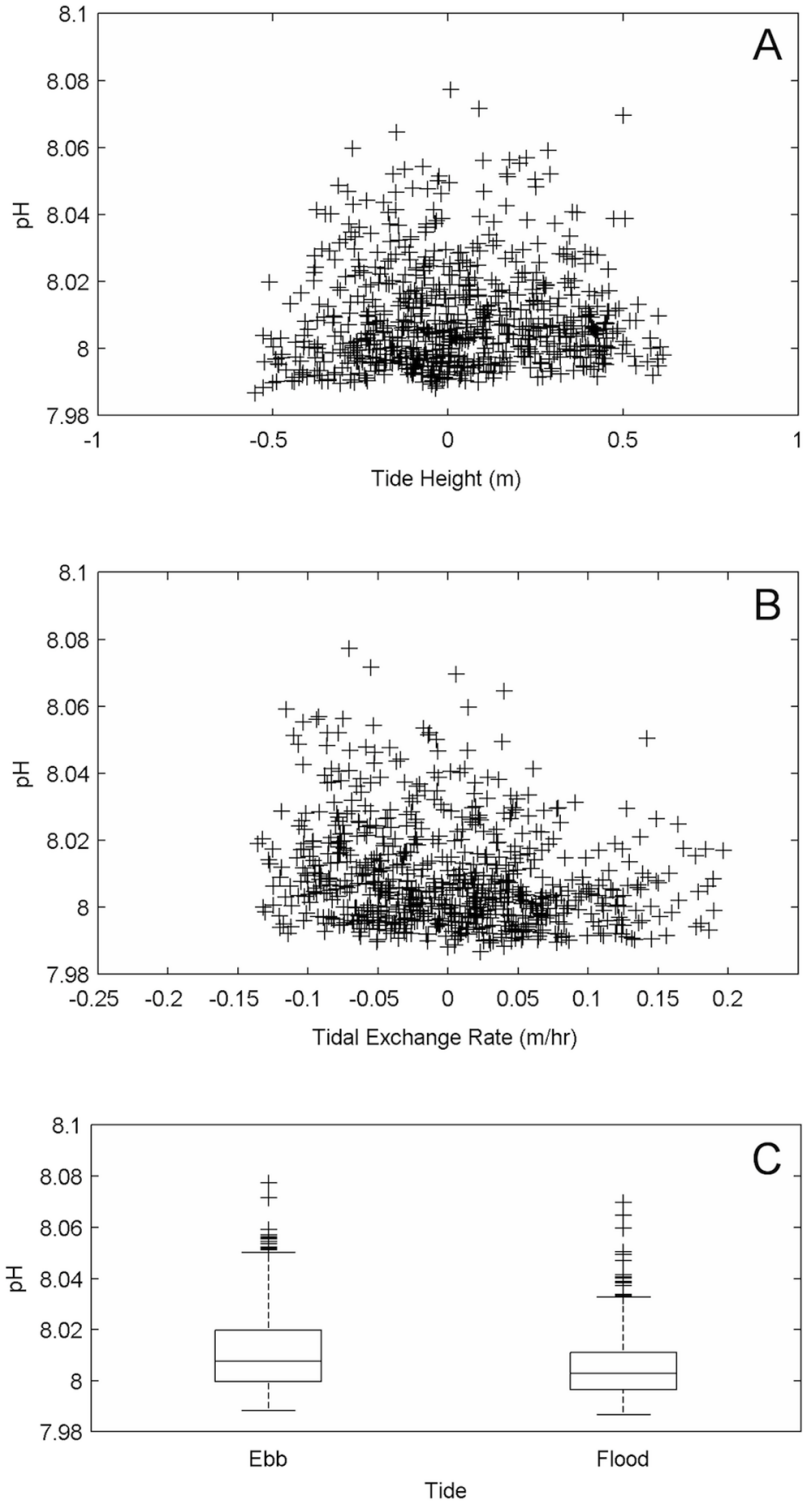
Relationship between pH and tides observed at Cape Evans. Panels show pH and tidal height (A), tidal exchange rate (B), and ebb versus flood stage (C).

Comparisons of pH spectra between Hut Point and Cape Evans indicate different dominant frequencies at each location. A large diel peak was present in the pH spectrum at Cape Evans, but not at Hut Point (Figure 5A); both exhibited variance increases at frequencies of 0.2 cpd and lower. Of the total variance within the pH time series at Cape Evans (0.00021), ∼25% occurs on a diel frequency (0.000052; variance integrated between 0.84 and 1.27 cycles per day). A similar diel peak is found in spectra of the tide and solar irradiance at Cape Evans (Figure 5B and 5C). While a relationship between pH and tide height was not apparent (Figure 6A), variation in pH appeared to increase marginally as tide exchange rate became more negative (Figure 6B). pH (mean ± s.d.) was greater during periods of ebb than flood flow (pH_ebb_: 8.0119±0.0161, *n = *397; pH_flood_: 8.0059±0.0135, *n = *371; Wilcoxon Rank Sum test: *W* = 125314, p<0.001; Figure 6C).

### Photosynthetic model outputs

**Figure 7:**
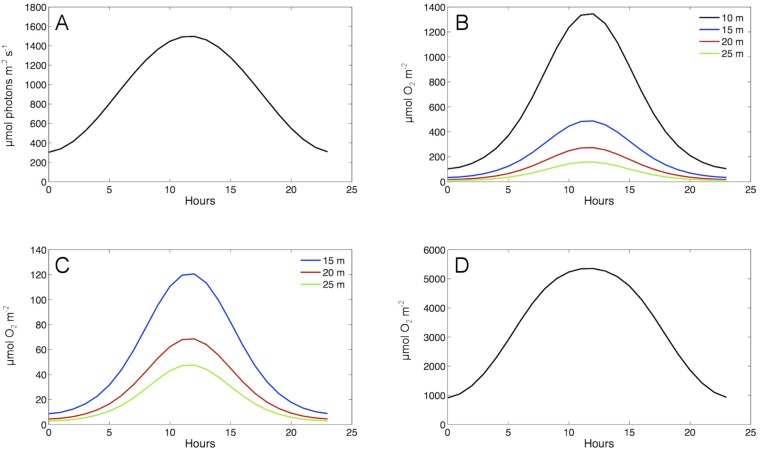
Model estimates of surface PAR (A) and hourly O_2_ production rate by algal functional groups over a 24 h period. Algal groups include foliose red (B), crustose coralline (C), and sea ice algae (D). Colored lines indicate estimated O_2_ production at different depths.

Based on observed values of surface PAR, Equation 1 was used to simulate hourly surface PAR for a 24 h period during late austral spring (mid November) with a mean of 900±433 µmol photons m^−2^ s^−1^ (mean ± s.d.), a maximum of ∼1500 µmol photons m^−2^ s^−1^, and a minimum of ∼300 µmol photons m^−2^ s^−1^ (Figure 7A). Equation 2 calculated 3.53% ±0.59 (mean ± s.d.) of surface PAR passed through 1.75 m of sea ice to be available for photosynthesis by sea ice algae. Based on an assumed sea ice algal biomass corresponding to 125 mg chl*a* m^−2^ (Table 2), light was further attenuated such that mean percentage of surface PAR available to benthic algae at depth over a 24 hr period was 0.060±0.023, 0.032±0.013, 0.017±0.0075, and 0.0092±0.0043, at 10 m, 15 m, 20 m, and 25 m, respectively.

**Table 2:** Photosynthesis-irradiance model parameters.

Parameter	Values [Source]
*k*_i_	1.35 m^−1^ [57]
*k*_m_	0.016 m^2^ mg^−1^ [57]
*k*_w_	0.09 m^−1^ [29]
*z*_i_	1.75 m
*z*_m_	125 mg chl*a* m^−2^ (mean value^1^)
*z*_w_	10–25 m [27], [29]
*P*_max, ia_	50.6 µmol O_2_ mg^−1^ chl*a* h^−1^ [42]
*P*_max, fr (net)_	6.3–10 µmol O_2_ gFW^−1^ h^−1^ [29]
*P*_max, cc (net)_	11.4–16.25 µmol O_2_ m^−2^ thallus h^−1^ [30]
*R*_ia_	3% of *P*_max_ [42]
α_ia_	1.03 µmol O_2_ mg^−1^ chl*a* h^−1^ (µmol photons m^−2^ s^−1^)^−1^ [42]
α_fr_	1.09–1.67 µmol O_2_ gFW^−1^ h^−1^ (µmol photons m^−2^ s^−1^)^−1^ [29]
α_cc_	4.6 µmol O_2_ m^−2^ thallus h^−1^ (µmol photons m^−2^ s^−1^)^−1^ [30]
*b*_ia_	same as z_m_
*b*_fr_	518–559 gFW m^−2^ [27], [29]
*b*_cc_	80–100% cover m^−2^ [27], [30]
TA	2341.2 µmol kgSW^−1^ (This study)
C_T_	2247.5 µmol kgSW^−1^ (This study)

Algal-specific values are presented for sea ice algae (ia), *Phyllophora antarctica* (fr), and crustose coralline (cc).

1 Mean value calculated from sea ice algae chlorophyll *a* measurements by [34], [43], [44], [69] at Cape Evans.

**Figure 8:**
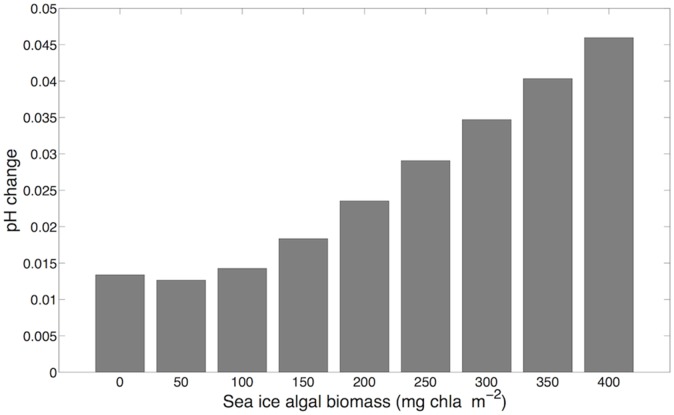
Model estimates of pH effect due to photosynthesis by algal community over a 24 h period in relation to sea ice algal biomass.

Model estimates of oxygen production rates differed greatly between the three algal groups (Figure 7). Based on parameters of algal biomass (Table 2), the maximum oxygen production rate by sea ice algae (5353 µmol O_2_ m^−2^ h^−1^; Figure 7D) was approximately 4 times greater than the highest estimated rate for foliose red algae (1345 µmol O_2_ m^−2^ h^−1^; Figure 7B) and 44 times greater than for crustose coralline red algae (121 µmol O_2_ m^−2^ h^−1^; Figure 7C). When averaged across depths (10 to 25 m), these oxygen production rates translate to a carbon drawdown of 5.34 µmol C kgSW^−1^ d^−1^ from the water column via algal photosynthesis. This removal of inorganic carbon, assumed to be entirely in the form of CO_2 (aq)_, would result in an increase of 0.016 pH units. When sea ice algal biomass was varied between 0 (no sea ice algae) and 400 mg chl*a* m^−2^ (theoretical maximum possible biomass [58]), pH increased between 0.013 to 0.046 units, respectively (Figure 8). While this pH effect range accounts for 14–51% of the maximum pH variation observed at Cape Evans, 95% of the diel variation in pH ranged 0.028 units, well within the model estimates. Analyses indicated that pH effects estimated by this model are most sensitive to changes in both light availability and carbonate system parameters (Table 3). Variation in the thickness and attenuation of sea ice doubled the effect on estimated pH due to photosynthesis. The greatest sensitivities were in the carbonate system via changes in alkalinity and total carbon; increasing TA increased the buffering potential within the system while decreases in C_T_ reduced the abundance of carbonate ion species (HCO_3_^−^ and CO_3_^−2^).

**Table 3:** Sensitivity analyses of model parameters for the light environment, algal biomass, and ocean carbonate system.

Parameter	Null+5%	Null−5%
sPAR	3.12	−3.91
*k*_i_	−11.72	11.72
*z*_i_	−11.72	11.72
*k*_m_	−1.56	1.56
*k*_w_	−0.78	0.78
*b*_ia_	3.12	−3.13
*b*_fr_	0	0
TA	−30.47	14.84
C_T_	11.72	−28.13

Values represent the percent change in estimated pH in response to an increase/decrease of each parameter value by 5%. Null value of each parameter is listed in Table 2.

## Discussion

This study deployed oceanographic sensors to observe high frequency variation in pH and water mass properties under fast sea ice at multiple locations within McMurdo Sound in the southern Ross Sea during austral spring, 2010. In addition, the potential for algal photosynthesis to serve as a biological driver of diel pH variation was estimated using a mechanistic model. The salient findings of this study were: (1) near-surface pH variation differed spatially between the two study locations with greater variation observed at the shallow coastal site than at the deeper location: (2) advection and water mass mixing were observed at Hut Point but did not appear to affect pH; and (3) modeling results suggest that under-ice algal photosynthesis is capable of driving a large portion of the diel pH variance observed at our shallow coastal site.

Based on the data presented here, near-surface waters (20 m depth) in McMurdo Sound experienced low levels of diel pH variation during this time period. The greatest pH range was observed at our shallow coastal location (Cape Evans) with a range of 0.0906 units, which is approximately 4–5 times lower than observed along the California coast [2]. These low levels of variation are similar to those first reported by [59] in McMurdo Sound during austral spring in 2010. Together, these observations support the expectation that Antarctic species living under fast sea ice are less likely to experience large fluctuations in pH over short time scales, unlike species found within upwelling systems [2], [60]. However, it is important to note that these observations are limited to spring. The transition from winter to summer is marked by a dramatic change in water column structure [5] as well as the delivery of the phytoplankton bloom from the Ross Sea polynya, north of Ross Island. The bloom, primarily consisting of *Phaeocystis pouchetti*, is transported under the sea ice in eastern McMurdo Sound by the southward flowing current and generally appears off McMurdo Station by early to mid December [61]. This bloom may be responsible for a seasonal shift of 0.3–0.5 pH units between winter and summer [4], [62]. Despite the relatively low pH variation, calcification conditions (i.e. saturation state, Ω) were still low, especially compared to both temperate [60] and tropical regions [63]. Longer duration deployments, ideally overwintering, are needed in order to capture the scale of high-frequency variation that occurs during this seasonal shift in productivity.

In addition to developing high-frequency time series of ocean pH under Antarctic sea ice, this study investigated whether abiotic or biotic processes may be driving the observed pH variation. Despite detecting at least three different water masses across our study locations, near-surface mixing events did not appear to contribute to the pH variation observed in McMurdo Sound. Temperature-salinity properties indicate that two water masses with properties similar to HSSW were present at Cape Evans. A water mass with properties similar to ISW was present continuously at New Harbor, while repeated mixing events between water masses were observed at Hut Point. It is possible that these events were driven by changes in near-surface flow direction, which has been shown to vary from southward to northward depending on the tidal phase during this time of year in eastern McMurdo Sound [24]. Further, yearlong mooring-based observations by [5] at Cape Armitage found the highest levels of variation in salinity and flow to occur at shallow depths (50–55 m versus 150–325 m). Salinity values were consistently lower (ΔS = 0.2–0.3) in this study compared to values reported by [20] but comparable to measurements by [5]. This may be attributed to the shallower sensor depth in this study (stationary at 20 m vs. vertical profiles from 0–600 m for [20]); the relative differences in salinity between the western and eastern Sound were consistent with those previously reported [20], [24]. The slight warming that occurs between Hut Point and New Harbor is likely due to the proximity of Hut Point to the Ross Ice Shelf [20]. The lack of a pH difference between the two primary water masses is interesting given their history of atmospheric contact. While HSSW flowing south from the Ross Sea polynya past Cape Evans at current speeds of ∼2–3 cm/s near the surface [21], [24] may have more recent atmospheric contact, ISW has very little, if any, contact with the atmosphere while underneath the Ross Ice Shelf. Modeling efforts estimate that 2.4–3.5 years is required for the formation of ISW from HSSW under the Ross Ice Shelf [64], [65], which in some places may be up to 700 m thick. Despite this difference in time under the ice between HSSW and ISW, median pH differed by only 0.0146 units between Hut Point and Cape Evans, and was slightly greater at Hut Point. Future deployments of pH sensors and current meters at multiple depths across McMurdo Sound and under the Ross Ice Shelf would add insight regarding processes affecting the carbonate system within these water masses.

Spatial differences in pH within McMurdo Sound appear to be more likely driven by a combination of biological production, bathymetry, and tidal exchange. The combination of the positive diel peak in pH with the increased pH during ebb tide periods at Cape Evans suggests the potential for pH variation in McMurdo Sound to be driven through a combination of photosynthetic production and tidal exchange between shallow and deep water columns. Sea ice algal biomass appeared to have a greater influence on increasing pH relative to benthic algae under mean levels of biomass, likely due to the higher quantity of available light at that depth. This community of microscopic algae appears to be patchy both across space and time [31], [33], [34], [66] making quantification of standing stock difficult over large areas. However, regional patterns have been reported with biomass tending to increase under thicker sea ice [67] and generally greater biomass in the western versus eastern McMurdo Sound [31]. Composition of the sea ice algal community appears to fluctuate seasonally, with shade-tolerant species being replaced as light levels increase through the summer [33]. The effect of these shifts in species assemblages on carbon drawdown and pH is not known, though increased levels of production would likely increase diel pH changes. Elevated pCO_2_ concentrations have been shown to increase growth of sea ice algae present in McMurdo Sound, though this effect is lost when pH drops below 7.6 [13]. Bathymetry may also be an important contributor to spatial difference in pH variation within McMurdo Sound. While the presence of sea ice algae is not depth dependent, their influence on dissolved inorganic carbon in the surrounding water is diluted as water column depth increases. This may help explain the relative lack of high-frequency pH variation observed at Hut Point, though the cause of the low-frequency decrease in pH is less clear. In addition to a deeper water column, Hut Point also had a thick layer of snow on the ice surface (>0.3 m), which would severely reduce the light available to sea ice algae. This spatial relationship between shallow versus deep locations was also seen in 2010, with greater diel variation observed at shallower locations (Cape Evans and Cinder Cones) than at a deeper site north of Hut Point (Erebus Basin) [59]. Tidal exchange may also play an important role in the observed pH variation. Previous work by [5], [21], [23], [24] showed spatial heterogeneity on periodic flow patterns (along-shore versus cross-shore) in areas of McMurdo Sound farther south of Cape Evans. If cross-shore transport were sufficient at Cape Evans, the increased pH variation observed during periods of ebb flow may be a signal of the shallow water moving past the mooring to deeper water. However, given the lack of such measurements in this study, it is not possible to test this hypothesis here. With lower levels of pH variation occurring over areas with greater depths, pelagic species may be less likely to experience natural fluctuations in pH on a regular basis, unlike species found in shallow coastal habitats. Further organismal research will be required to determine if these spatial differences in pH variation have led to differential tolerances and physiological responses in species residing in these two habitats.

This model showed daily photosynthetic production could account for a large proportion of the observed pH variance over a 24-hr period, but was unable to resolve temporal patterns in pH variation across subsequent days. This disconnection resembles that found by [68] when modeling pCO_2_ and O_2_ dynamics within ice-covered lakes; they suggest this is due to convective mixing under surface ice. The model presented here assumes a well-mixed water column, a common condition at Cape Evans during this time of year [27], [34], [43]. The magnitude of the pH effect may be reduced due to the formation of a boundary layer along the bottom of the sea ice, which may decrease the photosynthetic exchange of CO_2_ and O_2_ in the water column. This layer thickens under slower current speeds, reducing gas diffusion away from the sea ice algae [43]. As such, this physical process should be incorporated into future models, as well as additional parameters to account for tidal exchange and non-algal community respiration. Further, given the sensitivity of the model to variation in carbonate system parameters (alkalinity and C_T_), the effects of productivity on alkalinity and carbon species uptake should be included as it becomes available. These parameters could be empirically constrained by implementing repeated depth-stratified water sampling at regular intervals during a 24-h period, similar to the approach used by [53] for carbonate system effects of a *Phaeocystis* bloom in the Ross Sea polynya. Despite these limitations, this model offers a starting point to begin to explore a biological driver of pH variation in a unique environment that is threatened by changing global climate.
